# Completion of tuberculosis preventive therapy and associated factors among clients on antiretroviral therapy at Debre Berhan town health facilities, North Shoa Zone, Ethiopia

**DOI:** 10.1186/s12981-024-00629-0

**Published:** 2024-06-25

**Authors:** Alebachew Zewdu Tegegnework, Muluken Tessema Aemiro, Awraris Hailu Bilchut, Abinet Dagnaw Mekuria, Sisay Shewasinad Yehualashet

**Affiliations:** 1 HIV/AIDS Care Unit, Debre Berhan comprehensive Specialized Hospital, Amhara Regional State, Debre Berhan, Ethiopia; 2 School of Public Health, Asrat Woldeyes Health Science Campus, Debre Birhan University, Debre Birhan, Amhara Regional State, Debre Berhan, Ethiopia; 3 School of Nursing and Midwifery, Department of Paediatrics and Child Health Nursing, Debre Birhan University, Asrat Woldeyes Health Science Campus, Debre Birhan, Amhara Regional State, Debre Berhan, Ethiopia

**Keywords:** Tuberculosis preventive therapy, Antiretroviral therapy, Completion rate, Associated factors, Debre Berhan town, North Shoa Zone, Ethiopia

## Abstract

**Background:**

Tuberculosis preventive therapy is vital in caring for HIV-positive individuals, as it prevents the progression from latent tuberculosis infection to tuberculosis disease. The aim of the study is to assess the completion of tuberculosis preventive therapy and associated factors among clients receiving antiretroviral therapy in Debre Berhan town, Ethiopia, in 2022.

**Method:**

Institutional based cross sectional study was conducted. Random sampling methods were used to select both study participants and health facilities. Both bivariate and multivariate logistic regression analyses were performed. P-values less than 0.05 were statistically significant.

**Result:**

The study found that, 83% of participants were completed tuberculosis preventive therapy. Completed tuberculosis preventive therapy was associated with no adverse drug events, taking first-line ART, and good ART adherence.

**Conclusion:**

According to the Ethiopian ART guidelines, the study found a low completion rate of tuberculosis preventive therapy among HIV-positive clients on antiretroviral therapy. Factors like no adverse drug events, first-line antiretroviral regimen, and good adherence were significantly associated with completing tuberculosis preventive therapy.

**Supplementary Information:**

The online version contains supplementary material available at 10.1186/s12981-024-00629-0.

## Introduction

Tuberculosis (TB) is a major cause of death in people with human immunodeficiency virus (HIV) [1]. In 2019, 10 million people developed TB globally, leading to 1.4 million deaths, with 208,000 HIV-positive TB patients dying from TB-related causes [2, 3]. TB preventive therapy (TPT) is vital for reducing the TB burden in people living with HIV (PLHIV) [4]. Individuals with advanced HIV infection have a 20–37 times greater risk of developing TB compared to those without HIV [5].

In 2020, Ethiopia’s TB incidence rate was 140 per 100,000, with a TB death rate of 19 per 100,000 [2].

Ethiopia faces a dual HIV/AIDS and TB epidemic, underscoring the need for comprehensive strategies to tackle these interlinked diseases [6]. World Health Organization (WHO) recommends TB/HIV collaboration actions, including isoniazid preventive therapy (IPT) and antiretroviral therapy (ART), to provide integrated care and reduce TB burden among people living with HIV [7].

No study has been conducted in Debre Berhan town regarding completion of TPT among HIV-positive clients. Therefore, this study on TPT completion and associated factors can offer valuable insights for policymakers and health departments. It has the potential to inform evidence-based policies, assess existing programs, and guide the development of targeted interventions to prevent tuberculosis and enhance public health outcomes.

## Methods

An institutional based cross sectional study was conducted from June 1 to July 30, 2022 through chart reviews of HIV patients attending the ART clinic. The questionnaire was adapted and modified from previous literature [7–10]. Two health facilities: Debre Berhan Comprehensive Specialized Hospital and Debre Berhan Health Center were selected using simple random sampling from the 5 health facilities that provided ART services. From a total of 2,877 ART clients, 598 participants were selected using systematic sampling techniques. A pre-test was conducted on 5% of the total sample size at Chacha Health Center. Completeness, accuracy and consistency of the collected data were checked on daily basis.

The collected data was coded and entered into Epi Data version 3.1 software. Then, data were exported to SPSS version 22 for data analysis. Descriptive statistics was computed and the result was reported using frequencies and percentages. Multicollinearity test was conducted to assess the presence of collinearity among explanatory variables using the Variance Inflation Factor with (Max VIF = 1.29, Min VIF = 1.01). The adequacy of the final model was checked by Hosmer-Lemeshow test and it showed that the model was a good fit (P-value = 0.08). Bivariable and multivariable logistic regression were used to identify associated variables. Variable having p value < 0.25 in bivariable analysis was entered in to multi variable logistic regression model with 95% confidence interval and 5% significant level. Finally, in the multivariable logistic regression model, those variables that had significant association with the outcome variable at p-value less than 0.05 were declared as statistically significant.

## Result

The study had 581 participants, with a median age of 36 (21–51) years. Around 416 (71.6%) of the participants were male. 250 (43%) were married. 477 (82.1%) of the study participants were from an urban residence. 175 (30.1%) of the study participants had a college level education or higher. 258 (44.4%) of the participants were private sector workers. 359 (61.8%) had more than 2 family members. 229 (39.4%) of the study participants had a monthly income less than 35$ (supplementary file table: 1).

### Clinical characteristics

The study participants had the following characteristics: 492 (84.7%) had a functional status, 526 (90.5%) had good ART adherence and 468 (80.5%) did not experience any adverse drug events. The median CD4 count was 450 (SD ± 190.7), ranging from 45 to 995, with 322 (55.4%) having a CD4 count below 450.

### Prevalence of completion of tuberculosis preventive therapy

Approximately 484 participants (83%) successfully completed tuberculosis preventive therapy. Among the total participants, 158 individuals (27.2%) experienced adverse drug reactions related to TPT. Specifically, 77 participants (13.3%) reported nausea, while 27 participants (4.6%) developed a skin rash. All study participants were on ART medication, with 459 (79.1%) on a first-line regimen.

### Factors associated with completion of TPT

The odds of completing TPT were approximately nineteen times higher for clients aged 25–49 compared to clients aged 50 or older (AOR = 19.01, 95%CI: 8.36–43.23). Moreover, married clients had approximately four times higher odds of completing TPT compared to widowed clients (AOR = 4.51, 95%CI: 1.89–10.78). Clients who had not experienced adverse drug events had approximately three times higher odds of completing TPT compared to clients who had experienced such events (AOR = 3.52, 95% CI: 1.91–6.49).

Clients who were taking a first-line ART regimen had approximately two times higher odds of completing TPT compared to clients who were taking a second-line ART regimen (AOR = 2.60, 95% CI: 1.27–5.29). Clients who had good adherence to ART had approximately ten times higher odds of completing TPT compared to clients with poor adherence (AOR = 10.38, 95%CI: 4.14–25.96) (supplementary file table: 2).

## Discussion

The findings of this study provide important insights into the implementation of TB preventive treatment among people living with HIV in the local healthcare setting. The overall TPT completion rate was 83% (95% CI: 80.2–86.1%), which was slightly below the 90% target recommended by the WHO [11]. This suggests that while the program is performing reasonably well, there is room for improvement to achieve the global TPT coverage goals. Similar findings were observed in other studies conducted in Zimbabwe (81%) and Malaysia (86%) [12, 13]. However, the completion rate in this study was lower compared to that reported in a retrospective study conducted in the western region of Nepal (94.3%) and a cross-sectional study conducted in Swaziland (89.4%) [7, 14]. Furthermore, the completion rate in this study was higher when compared to the rates reported in a cross-sectional study conducted in Tigrai, Ethiopia (62.1%), a cross-sectional study conducted in Kenya’s national referral hospital (82%), and a prospective cohort study conducted in Thailand (69%) [8, 9, 15].

In this study majority of participants 526 (90.5%) had good adherence level to ART. Comparing these findings to similar studies conducted in different locations, it was noted that the adherence level in this study was almost similar those reported in previous studies conducted in Addis Ababa, Gondar town (90.3%), and Swaziland (94.8%) [14, 16, 17]. However, the adherence level in this study was higher when compared to a study conducted in Tigrai, where only 62.3% of the participants demonstrated good adherence [8]. A closer examination of the results reveals potential drivers and barriers to TPT completion in this context. Patients with good adherence to antiretroviral therapy were significantly more likely to also complete TPT (adjusted OR: 2.31, 95% CI: 1.47–3.63). This alignment of adherence behaviors may be attributable to the counseling and support services provided through the integrated HIV/TB program. Patients receiving coordinated messaging and follow-up for both their ART and TPT regimens may be more motivated and empowered to adhere to both treatments [18]. Additionally, the healthcare system’s practice of synchronizing ART and TPT medication refills could facilitate adherence by minimizing the burden on patients [19].

In contrast, TPT completion was lower among patients who were unemployed (adjusted OR: 0.47, 95% CI: 0.30–0.74) or had advanced HIV disease (adjusted OR: 0.48, 95% CI: 0.31–0.75). These socioeconomic and clinical factors likely introduce practical and psychological barriers that impede consistent medication-taking behaviors [20, 21]. Patients with limited financial resources or more advanced illness may face greater challenges in regularly accessing healthcare facilities, affording transportation, and sustaining the motivation required for prolonged preventive treatment.

The study also found that patients diagnosed with TB during TPT had significantly lower completion rates (adjusted OR: 0.22, 95% CI: 0.13–0.37). This is concerning, as these individuals are at heightened risk for poor outcomes and may require more intensive monitoring and support to successfully complete their treatment [22]. Potential contributors to the lower completion in this group could include higher rates of treatment interruptions, adverse events, and deteriorating health status.

In these study clients who were taking a first-line ART regimen were more likely to complete their TPT medication compared to clients who were taking a second-line ART regimen. Clients on the first-line ART regimen were 2.6 times more likely to complete TPT compared to those on the second-line regimen. In this study 459(79.1%) participants were on a first-line ART regimen. These findings were lower than studies conducted in South India (84.6%) and the Far Western Region of Nepal (85.6%) [7, 23].

### Limitations of the study

The accuracy and completeness of the data recorded in the medical charts were varying. Cross-sectional studies capture data at a single time point, which makes it difficult to establish temporal relationships or determine causality between variables.

## Conclusion

According to the Ethiopian ART guidelines, the study found a low completion rate of tuberculosis preventive therapy among HIV-positive clients on antiretroviral therapy. Based on the findings, the following recommendations are suggested for different stakeholders, the findings of this study underscore the need for targeted interventions to strengthen TPT adherence, particularly among clients on second-line ART regimens. Reinforcing counseling, streamlining medication access, and providing additional resources for vulnerable populations may help address the barriers identified in this setting. Careful tracking of TPT outcomes, particularly among high-risk groups, will also be critical to continuously improve program performance and achieve the global targets for TPT coverage and completion.

### Electronic supplementary material

Below is the link to the electronic supplementary material.

Supplementary Material 1

## Data Availability

The data that support the findings of this study are available upon reasonable request without restrictions from the corresponding authors Alebachew Zewdu Tegegnework and Sisay Shewasinad Yehualashet. Any additional data related to this study can be made available upon request to the corresponding authors.

## Abbreviations

AIDS: Acquired Immune Deficiency Syndrome
ART: Anti-Retro Viral Therapy
HIV: Human Immune Deficiency Virus
IPT: Isoniazid Preventive Therapy
PLWHA: People Living With HIV/AIDS
TB: Tuberculosis
TPT: Tuberculosis Preventive Therapy
WHO: World Health Organization
