# Supplementary material for: Completion of tuberculosis preventive therapy and associated factors among clients on antiretroviral therapy at Debre Berhan town health facilities, North Shoa Zone, Ethiopia
Source: AIDS Res Ther. 2024 Jun 25;21:44. doi: 10.1186/s12981-024-00629-0 (PMC11197169; doi:10.1186/s12981-024-00629-0)
Supplement: Supplementary file 1 — Supplementary Material 1 [file 12981_2024_629_MOESM1_ESM.docx]

**Supplementary Data**

Table 1: Socio-demographic characteristics of the study participants

| Variables | Categories | Frequencies | Percentages |
| --- | --- | --- | --- |
| Age in years | < 25 | 74 | 12.8 |
|  | 25–49 | 318 | 54.7 |
|  | > 50 | 189 | 32.5 |
| Sex | Male | 416 | 71.6 |
|  | Female | 165 | 28.4 |
| Marital status | Single | 110 | 18.9 |
|  | Married | 250 | 43.0 |
|  | Divorced | 151 | 26.1 |
|  | Widowed | 70 | 12.0 |
| Religion | Orthodox | 493 | 84.9 |
|  | Muslim | 25 | 4.3 |
|  | Protestant | 63 | 10.8 |
| Residence | Urban | 477 | 82.1 |
|  | Rural | 104 | 17.9 |
| Educational status | Don’t read and write | 61 | 10.5 |
|  | Only read and write | 97 | 16.7 |
|  | Primary school | 94 | 16.2 |
|  | Secondary school | 154 | 26.5 |
|  | College and above | 175 | 30.1 |
| Main occupation | Private worker | 258 | 44.4 |
|  | Merchant | 142 | 24.4 |
|  | Gov’t employee | 98 | 16.9 |
|  | House wife | 46 | 7.9 |
|  | Student | 37 | 6.4 |
| Family number | <=2 | 222 | 38.2 |
|  | 3–5 | 359 | 61.8 |
| Monthly income (USD) | <$35 | 229 | 39.4 |
|  | $35-$70 | 167 | 28.7 |
|  | >=$70 | 185 | 31.9 |

Table 2: Factors Associated with TPT Completion.

| Variables | Categories | Complete TPT | Incomplete TPT | COR | AOR | P-Value |
| --- | --- | --- | --- | --- | --- | --- |
| Age | < 25 | 49(66.2%) | 25(33.8%) | 0.7(0.4–1.3) | 1.43(0.70–2.93) | 0.318 |
|  | 25–49 | 296(93.1%) | 22(6.9%) | 4.8(2.8–8.3) | **19.01(8.36–43.2)** | **0.0001**** |
|  | >=50 | 139(73.5%) | 50(26.5%) | 1 | 1 |  |
| Marital status statusstatus | Single | 91(82.7%) | 19(17.3%) | 1.9(0.9–3.7) | 1.98(0.76–5.37) | 0.175 |
|  | Married | 225(90.0%) | 25(10.0%) | 3.4(1.7–6.6) | **4.51(1.89–10.78)** | **0.001**** |
|  | Divorced | 117(77.5%) | 34(22.5%) | 1.3(0.7–2.5) | 1.60(0.70–3.66) | 0.264 |
|  | Widowed | 51(72.9%) | 19(27.1%) | 1 | 1 |  |
| Educational status | Don’t read and write | 42(68.9%) | 19(31.1%) | 1 | 1 |  |
|  | Read and write | 86(88.7%) | 11(11.3%) | 3.5(1.5–8.1) | 2.6 (0.88–7.65) | 0.082 |
|  | Primary school | 71(75.5%) | 23(24.5%) | 1.4(0.68–2.7) | 0.85(0.32–2.22) | 0.741 |
|  | Secondary school | 125(81.2%) | 29(18.8%) | 1.9(0.99–3.8) | 1.32(0.50–3.44) | 0.565 |
|  | College and above | 160(91.4%) | 15(8.6%) | 4.8(2.3–10.3) | 2.07(0.73–5.81) | 0.167 |
| Main occupation | Gov’t | 92(93.9%) | 6(6.1%) | 4.23(1.4–13) | 1.6(0.3–7.9) | 0.571 |
|  | Private | 207(80.2%) | 51(19.8%) | 1.12(0.5–2.6) | 1.2(0.4–3.9) | 0.756 |
|  | House wife | 37(80.4%) | 9(19.6%) | 1.13(0.4–3.3) | 0.7(0.2–3.3) | 0.663 |
|  | Merchant | 119(83.8%) | 23(16.2%) | 1.42(0.6–3.5) | 1.3(0.4–4.6) | 0.665 |
|  | Student | 29(78.4%) | 8(21.6%) | 1 | 1 |  |
| Monthly income (USD) | < $35 | 178(77.7%) | 51(22.3%) | 1 | 1 |  |
|  | $35-$70 | 139(83.2%) | 28(16.8%) | 1.42(0.9–2.4) | 1.2(0.6–2.3) | 0.676 |
|  | >=$70 | 167(90.3%) | 18(9.7%) | 2.65(1.5–4.7) | 1.1(0.5–2.5) | 0.858 |
| Drug adverse effect  adverse ADE related to TPT | No | 404(86.3%) | 64(13.7%) | 2.60(1.6–4.2) | **3.5(1.9–6.5)** | **0.0001**** |
|  | Yes | 80(70.8%) | 33(29.2%) | 1 | 1 |  |
| ART regimen | 1st line | 396(86.3%) | 63(13.7%) | 2.42(1.5–3.9) | **2.6(1.3–5.3)** | **0.008**** |
|  | 2nd line | 88(72.1%) | 34(27.9%) | 1 | 1 |  |
| Adherence to ART | Good | 453(86.1%) | 73(13.9%) | 4.80(2.7–8.6) | **10.4(4–26)** | **0.0001**** |
|  | Poor | 31(56.4%) | 24(43.6%) | 1 | 1 |  |

AOR = adjusted odds ratio; COR = crude odds ratio; CI = confidence interval, ****** *is* for variables with p < 0.05
